# Risk factors for ischemic stroke; results from 9 years of follow-up in a population based cohort of Iran

**DOI:** 10.1186/1471-2377-12-117

**Published:** 2012-10-02

**Authors:** Noushin Fahimfar, Davood Khalili, Reza Mohebi, Fereidoun Azizi, Farzad Hadaegh

**Affiliations:** 1 Prevention of Metabolic Disorders Research Center, Research Institute for Endocrine Sciences, Shahid Beheshti University of Medical Sciences, P.O. Box 19395-4763, Tehran, Islamic Republic of Iran; 2 Department of epidemiology, School of public health, Shahid Beheshti University of Medical Sciences, Tehran, Iran; 3 Endocrine Research Center, Research Institute for Endocrine Sciences, Shahid Beheshti University of Medical Sciences, Tehran, Iran

**Keywords:** Chronic kidney disease, Hypertension, Diabetes, Stroke, Population attributable fraction

## Abstract

**Background:**

Data about the risk factors of stroke are sparse in the Middle East populations. We aimed to determine the potential risk factors and their population attributable fraction (PAF) for stroke in an Iranian population.

**Methods:**

A cohort Study consisted of 1089 men and 1289 women, with mean (SD) ages of 61.1(7.6) and 59.0(6.7) years, respectively. Cox regression was implemented to estimate the hazard ratio (HR) of each risk factor for stroke events in a stepwise method. We calculated a multivariate adjusted population attributable fraction (PAF) for any risk factors remained in the model.

**Results:**

During 9.3 years of follow-up, 69 events of stroke occurred with incidence rates of 4.5 (95% CI: 3.3-6.0) and 2.5 (1.7-3.6) in 1000 person-years for men and women respectively. Among potential risk factors, only age ≥ 65 years (HR: 2.03, CI: 1.24-3.31), male gender (HR: 2.00, CI: 1.16-3.43), hypertension (HR: 3.03, CI: 1.76-5.22), diabetes mellitus (HR: 2.18, CI: 1.34-3.56), and chronic kidney disease (CKD) (HR: 2.01, CI: 1.22-3.33), were independently associated with increased risk of stroke events in the total population. A paired homogeneity test showed that the hazard ratio of CKD did not differ from other independent risk factors. The PAFs were 29.7% and 25% for male gender and age ≥ 65 as non-modifiable and 48.6%, 29.1% and 22.0% for hypertension, CKD and diabetes as modifiable risk factors respectively.

**Conclusion:**

Following this population based study of Iranians, we demonstrated that among modifiable risk factors, CKD as well as hypertension and diabetes are the strongest independent predictors of stroke.

## Background

Stroke is the second leading cause of death worldwide and the leading cause of functional impairments in adults which could affects both patients and their relatives in most regions [1,2]. As populations are getting older, a dramatic increase in prevalence and burden of disability is expected in the future. In the Middle East and North Africa (MENA), stroke is progressively becoming a major health problem and it is estimated that its current mortality will double by 2030 [3]. Incidence rate of stroke and its case fatality rate varies extensively among studies in this region [4]. In this respect, the prevention strategies need to be implemented urgently.

Prospective studies have revealed different risk factors for stroke subtypes [5]. The risk factors for ischemic stroke include aging, hypertension, diabetes, smoking, history of cardiovascular diseases (CVD), atrial fibrillation, and left ventricular hypertrophy [5,6]. The INTERSTROKE study, involving 22 countries demonstrated that ten risk factors account for 90% of the population-attributable risks for stroke events. However, not all potential risk factors were considered in INTERSTROKE study [7]. Recently, a meta-analysis highlighted the importance of the independent association between low glomerular filtration rate and incident stroke [8]. The study also demonstrated the stronger effect of chronic kidney disease (CKD) on stroke among Asian populations compared to non-Asian populations.

There is a scarcity of reliable information on the possible risk factors and population-attributable risks for stroke in the developing countries, especially in Middle East region [9]. Also, the incidence rates of regional stroke and measure of association need to be clarified to provide optimal treatment and reduce the burden of stroke. In this study, we aimed to determine the potential risk factors and their population attributable fraction (PAF) for stroke in an Iranian population during 9.3 years of follow-up.

## Methods

### Study population

Tehran Lipid and Glucose Study (TLGS) is a prospective population-based cohort study carried out on 15005 people aged, 3 years and over living in district-13 of Tehran, to ascertain the risk factors for non-communicable diseases. Subjects were divided into the cohort and intervention groups, the latter being instructed on how to modify their life style; the method of this educational intervention has been described elsewhere [10,11]. There were 3394 participants, aged ≥50 years, in the first phase of TLGS (February 1999 to August 2001). We excluded 435 participants with CVD history at baseline. After further excluding, 184 subjects with missing data at baseline and 347 subjects without any follow-up data, remained 2378 participants (80.4% of the eligible subjects;) who were followed until 20 March 2009 with a median follow up of 9.3 years. Data of all these participants including 953 individuals of the intervention group and 1425 individuals of the cohort group were pooled for final analysis.

The proposal of this study was approved by the ethical committee of the Research Institute for Endocrine Sciences and informed written consent were obtained from all subjects.

### Clinical and laboratory measurements at the baseline

All information was gathered by a trained interviewer, using a pretested questionnaire which included demographic data, past medical history, drug consumption and smoking behavior. Blood pressure and anthropometrical measurements were collected during physical examinations.

After 12 to 14 hours overnight fasting, a blood sample was taken and fasting plasma glucose (FPG), total cholesterol (TC), high density lipoprotein cholesterol (HDL-C), triglyceride (TG) and creatinine levels were measured on the day of blood sampling at the TLGS research laboratory. Two hour plasma glucose (2 h-PG) was also measured in participants without any known diabetes. The details of data gathering have been described before [10].

### Definition of terms

We defined the variables studied as follows: Diabetes mellitus (DM), FPG ≥7 mmol/l, 2 h-PG ≥11.1 mmol/l or current use of glucose lowering medication; pre-diabetes i.e. impaired fasting glucose (IFG) or impaired glucose tolerance (IGT), FPG ≥5.55 mmol/l or 2 h-PG ≥7.77 mmol/l in non-diabetic subjects; hypertension, systolic blood pressure ≥ 140 mmHg and/or diastolic blood pressure ≥ 90 mmHg or current use of antihypertensive medication; Pre-hypertension, SBP ≥120 mmHg or DBP ≥80 mmHg in non-hypertensive subjects; hypercholesterolemia, TC ≥6.20 mmol/l or using anti lipid drugs; Low HDL, HDL <1.03 mmol/l; high triglycerides, TG ≥2.258 mmol/l or anti-lipid drug use; general obesity, body mass index (BMI) ≥30 kg/m^2^; central obesity, waist circumference ≥95 cm in both genders for an Iranian population [12]; family history of premature CVD, any prior diagnosis of CVD in male and female first-degree relatives under 55 and 65 years, respectively; smoking, current or past use of cigarettes or other smokes(water-pipes, pipes); Intervention, participants who were educated to change life style [10].

According to guideline of the Kidney Disease Outcome Quality Initiative(KDOQI), chronic kidney disease is defined as either kidney damage or Glomerular Filtration Rate (GFR) <60 mL/min/1.73 m^2^ for >3 months [13]. For this study GFR was estimated using the abbreviated prediction equation, provided by the Modification of Diet in Renal Disease (MDRD) study as follows:

Abbreviated MDRD study equation:

$$\begin{array}{c} \text{GFR}=186\times {\left(\text{SCr}\right)}^{-1.154}\times {\left(\text{Age}\right)}^{-0.203}\\ \times \left(0.742\;\text{if female}\right) \end{array}$$

In this equation, eGFR (estimated GFR) is expressed as mL/min per 1.73 m^2^ and serum creatinine (SCr) is expressed as mg/dL [14].

Prevalent CKD was considered an eGFR lower than 60 mL/min/1.73 m^2^ occurring at baseline; this corresponds to stages 3 to 5 CKD based on the KDOQI guidelines.

### Definition of outcome

The data collection process on cardiovascular outcomes has been published in detail earlier [10]. To summarize, each year and in a continuous manner, a trained nurse questioned each participant for any medical event leading to hospitalization in the past year, using telephone call; if a related event had occurred, complementary data were collected by a trained physician during a home visit or a visit at the hospital. In the case of mortality, data were collected from the hospital (based on death certificate or the forensic medicine report) and if needed a verbal autopsy i.e. a method of finding as much data as possible about a dead person by asking questions of the close family and others who can explain the mode of death and conditions preceding death. Collected data were evaluated by an outcome committee consisting of a principal investigator, an internist, an endocrinologist, a cardiologist, an epidemiologist, the physician who collected the data and other invited experts, as needed.

Stroke was defined according to the WHO definition as, "rapidly developing clinical signs of or global disturbance of cerebral function, lasting > 24 hours or leading to death with no apparent cause other than that of vascular origin" [15]. Additionally, we used “imaging of an acute clinically relevant brain injury in patients with fast vanishing symptoms” as another criterion of definite stroke. Possible stroke was defined as onset of an acute focal neurological deficit with absent imaging that is indicative of stroke but for which there is insufficient data to establish whether the symptoms and the period fully qualify for the WHO definition for definite stroke [3]. Patients with Transient Ischemic Attack (TIA) may meet the above definition of possible stroke with the addition that their symptoms resolve within 24 hours. All cases, regardless of definite or possible stroke or TIA description were included in the cerebrovascular accident (CVA/stroke) definition. We classified strokes as ischemic or hemorrhagic according to all available information from informant interviews, medical records, and brain imaging studies (Computed Tomography and Magnetic Resonance Imaging).

### Statistics

Baseline characteristics for categorical variables were presented as percentages. Log-Rank test was used to test the equality of stroke free survival between the levels of these exposure variables.

Cox proportional hazard regression model was used to investigate the hazard ratio (HR) of each risk factor. Time to event was defined as time of censoring or having event, whichever came first. We censored individuals at the time of other causes of death, leaving the residence area and loss to follow up or being in the study until March 2009, without any CVA event. The proportional hazard assumption was tested graphically and using the time-dependent covariate test. All proportionality assumptions were generally appropriate. To detect the most important risk factors of CVA, a forward stepwise approach was used (p <0.2 for entry and p >0.1 for removal). The following covariates were used in the model: old age (> = 65 y.), sex, hypertension, DM, hypercholesterolemia, high TG, low HDL, general obesity, central obesity, family history of premature CVD, smoking and CKD; intervention was also considered as a possible confounder. A modified version of the Hosmer-Lemeshow chi-square for survival data was used to check the model fitness [16]. We calculated a multivariate adjusted population attributable fraction (PAF) for any risk factors that remained in the model [17]. A paired homogeneity test, which is a Wald test of the linear hypothesis of the Cox model regression coefficients, was performed to test the null hypothesis that the hazard ratio for CKD was equal to that for other risk factors in prediction of incident stroke. To be more specific, the Cox analysis was repeated for continuous data including age, SBP, DBP, FPG, TC, TG, HDL-C, BMI, waist circumference and eGFR besides sex, family history of premature CVD, smoking and intervention.

Additionally, to find out the role of pre-diabetes and pre-hypertension in predicting CVA, we compared the Kaplan-Meier failure rates of these categories.

To increase the study power, we pooled men and women, because, there was not significant interaction between sex and other risk factors related to CVA outcome.

All analyses were carried out using STATA version 10 and statistically significant level was defined as 2-tailed P-value below than 0.05.

## Results

The study sample consisted of 1089 men and 1289 women, with mean (SD) ages of 61.1 (7.6) and 59.0 (6.7) years, respectively. There was no significant difference between the followed and non-followed participants with regards to age and other major risk factors of CVA. During 9.3 years of follow-up, 69 (41 men) cases of stroke events occurred with an incidence rate of 4.5 (95% CI: 3.3-6.0) and 2.5 (1.7-3.6) in 1000 person-years of men and women respectively. Most of the cases were ischemic stroke and only 3 cases in women and 5 cases in men were hemorrhagic ones. Incidence rates of stroke according to the baseline exposure variables are summarized in Table 1. Log-Rank test showed that the stroke free survival was differed between levels of age, sex, hypertension, DM, smoking and CKD exposure variables. These variables, whether examined as continuous or binary, were the most important risk factors of CVA based on the stepwise Cox proportional hazard model as well (Table 2). Hypertension with a HR of 3.03 (1.76- 5.22) and smoking with HR of 1.63 (0.94- 2.80) had the highest and the lowest significant HRs respectively. The other significant risk factors had a HR of around two. Paired homogeneity test showed that the hazard ratio of CKD was not different from other independent risk factors (all p >0.2). The Hosmer-Lemeshow test was non-significant (p = 0.4) indicating goodness of fit for the model. The PAFs were 29.7% and 25% for male gender and age ≥ 65 as non-modifiable and 48.6%, 29.1% and 22.0% for hypertension, CKD and diabetes respectively as modifiable risk factors (Table 2). The results did not change when our analysis limited to those with ischemic stroke as the outcome (Table 3).

**Table 1:** Incidence rates of stroke according to the baseline exposure variables

**Exposure variable**	**No. (%) Total: 2378**	Incidence (95%CI) * Exposure +	Incidence (95%CI) * Exposure −	**P- Value†**
**Age, ≥ 65**	645 (27.1)	6.6 (4.7-9.2)	2.3 (1.7-3.2)	< 0.001
**Sex, male**	1089 (45.8)	4.5 (3.3-6.0)	2.5 (1.7 -3.6)	0.017
**Hypertension**	1057 (44.4)	5.6 (4.3 -7.4)	1.7 (1.1-2.6)	< 0.001
**Diabetes**	544 (22.9)	6.3 (4.4-9.1)	2.6 (1.9-3.5)	< 0.001
**Smoking**	548 (23.0)	5.1 (3.4-7.7)	2.9 (2.2-3.9)	0.020
**Obesity**	707 (29.7)	3.4 (2.2-5.2)	3.4 (2.5-4.5)	0.995
**High WC**	1121 (47.1)	4.1 (3.0-5.6)	2.8 (1.9-4.0)	0.102
**Low HDL-C**	1062 (44.7)	3.5 (2.5-5.0)	3.3 (2.4-4.5)	0.732
**Hypertriglyceridemia**	955 (40.2)	4.1 (3.0-5.8)	2.9 (2.1-4.0)	0.134
**Hypercholesterolemia**	1017 (42.8)	3.4 (2.4-4.9)	3.3 (2.4-4.6)	0.906
**Intervention**	953 (40.1)	3.4 (2.3-4.9)	3.4 (2.5-4.6)	0.987
**FH CVD**	363 (15.3)	4.2 (2.4 -7.2)	3.2 (2.5-4.2)	0.410
**CKD**	964 (40.5)	5.0 (3.6-6.8)	2.4 (1.6-3.4)	0.001

*Incidence density per 1000 person-years.

†Based on Log Rank test for equality of stroke free survival between exposure-positive and exposure-negative groups.

*CI*: confidence interval; *WC*: waist circumference; *HDL-C*: HDL cholesterol; Intervention: being in the intervention group; *FH CVD*: family history of premature cardiovascular disease; *CKD*: chronic kidney disease.

**Table 2:** Risk factors of stroke and their Population Attributable Fraction based on Cox proportional hazard model*

	**HR**	**95% CI**	**P-value**	**PAF (%)**
**Model including binary variables**				
**Age, ≥ 65 years**	2.03	(1.24- 3.31)	0.005	25.0
**Sex, male**	2.00	(1.16- 3.43)	0.012	29.7
**Smoking**	1.63	(0.94- 2.80)	0.081	12.9
**Hypertension**	3.03	(1.76- 5.22)	< 0.001	48.6
**Diabetes**	2.18	(1.34- 3.56)	0.002	22.0
**Chronic Kidney Disease**	2.01	(1.22- 3.33)	0.007	29.1
**Model including continuous variables**				
**Age, year**	1.08	(1.04-1.11)	< 0.001	
**Sex, male**	1.74	(1.02-2.98)	0.044	
**Smoking**	1.75	(1.02-3.01)	0.042	
**Systolic blood pressure, cmHg**	1.13	(1.00-1.28)	0.045	
**Diastolic blood pressure, cmHg**	1.55	(1.21-1.98)	0.001	
**Fasting plasma glucose, mmol/l**	1.10	(1.01-1.18)	0.021	
**GFR, mL/min/1.73 m2**	0.97	(0.95-0.99)	0.008	

* These risk factors remained in the model through a forward stepwise method; other variables (obesity, high waist circumference, low HDL cholesterol, hypertriglyceridemia, hypercholesterolemia, intervention and family history of premature cardiovascular disease) excluded from the model because of p < 0.2 for entry or p > 0.1 for removal. Hosmer-Lemeshow chi-square (modified version for survival data) was <20 indicating a good fitness of the model.

*HR*: Hazard Ratio; *CI*: Confidence Interval; *PAF*: Population Attributable Fraction; *GFR*: Glomerular Filtration Rate.

**Table 3:** Risk factors of Ischemic stroke and their Population Attributable Fraction based on Cox proportional hazard model*

	**HR**	**95% CI**	**P-value**	**PAF (%)**
**Model including binary variables**				
**Age, ≥ 65 years**	2.04	(1.21- 3.44)	0.007	24
**Sex, male**	1.93	(1.90- 3.43)	0.024	28.4
**Smoking**	1.73	(0.97- 3.08)	0.063	14.5
**Hypertension**	2.73	(1.55- 4.81)	0.001	44.7
**Diabetes**	2.38	(1.42- 3.99)	0.001	24.7
**Chronic Kidney Disease**	2.09	(1.22- 3.58)	0.007	30.8
**Model including continuous variables**				
**Age, years**	1.07	(1.03-1.11)	<0.001	
**Sex, male**	1.69	(0.95-3.00)	0.072	
**Smoking**	1.83	(1.04-3.25)	0.038	
**Systolic blood pressure, cmHg**	1.13	(0.99-1.28)	0.075	
**Diastolic blood pressure, cmHg**	1.51	(1.16-1.96)	0.002	
**Fasting plasma glucose, mmol/l**	1.11	(1.03-1.20)	0.009	
**GFR, mL/min/1.73 m2**	0.97	(0.94-0.99)	0.014	

* These risk factors remained in the model through a forward stepwise method; other variables (obesity, high waist circumference, low HDL cholesterol, hypertriglyceridemia, hypercholesterolemia, intervention and family history of premature cardiovascular disease)were excluded from the model because of p < 0.2 for entry or p > 0.1 for removal. Hosmer-Lemeshow chi-square (modified version for survival data) was <20 indicating a good fitness of the model.

*HR*: Hazard Ratio; *CI*: Confidence Interval; *PAF*: Population Attributable Fraction; *GFR*: Glomerular Filtration Rate.

**Figure 1:**
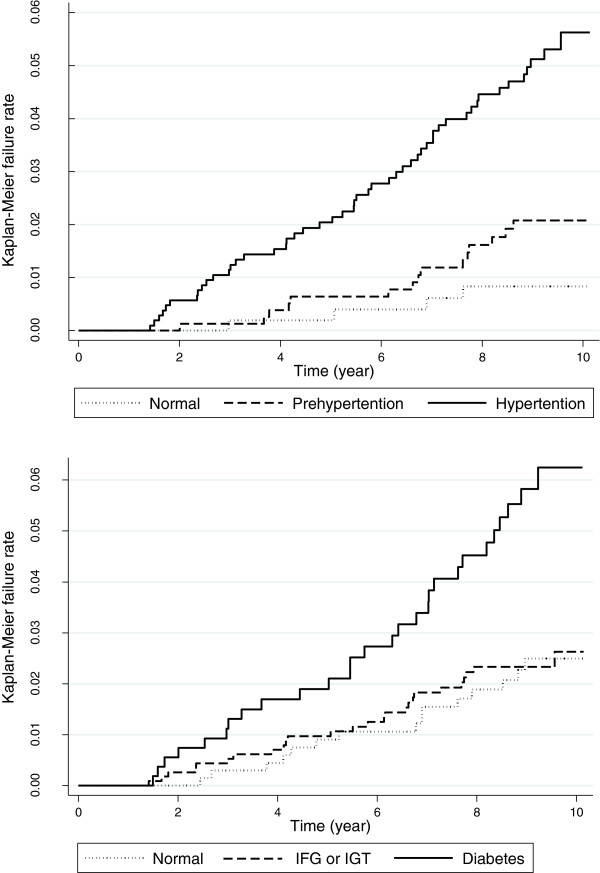
Incidence rates of stroke events regarding different categories of hypertension and glucose intolerance.

Kaplan-Meier failure rate and Log-Rank test showed no difference between pre-diabetic and non-diabetic subjects (p = 0.184) nor between pre-hypertensive and normotensive subjects (p = 0.102) regarding CVA outcome (Figure 1).

## Discussion

To the best of our knowledge, this is the first study conducted in the Middle East region reporting the population attributable fraction for stroke events. Results from this study suggest that most stroke events in Iranian populations can be attributed to modifiable risk factors of hypertension, CKD and diabetes. There was no difference between CKD, hypertension and diabetes, with regard to the prediction of total as well as ischemic stroke events.

The INTERSTROKE study was the first large case–control study aimed at ascertaining risk factors for stroke, in which low and middle income countries like Iran were included [7]. The study showed that five established risk factors (reported hypertension, smoking status, waist to hip ratio, diet risk score and physical activity) account for 80% cases of stroke events. With addition of five other risk factors (diabetes, alcohol intake, psychosocial factors, cardiac causes and ApoA1), the population-attributable risks for stroke reached 90%, a finding which indicated that there are some other risk factors that need to be considered beyond these potential risk factors, bearing in mind that usually the sum of PAFs goes beyond than 100% [18].

Since, CKD and CVD often shared the same pathophysiological mechanism [19] and most of those who had CKD died because of their CVD problems not progression to end stage renal failure, much attention is being paid to the magnitude of relationship between CKD and stroke [20]. In our dataset, we demonstrated the importance of CKD as the second strongest predictor of stroke. CKD, independent of age, gender, smoking, hypertension and diabetes, was associated with ischemic stroke and accounts for 30% of all stroke patients. Importantly, we found the same risk for CKD vs. other independent risk factors in prediction of incident stroke; obviously, the higher PAF of hypertension compared with CKD was related more to higher prevalence of the former than to the latter in our population. Recently in a meta-analysis of 21 articles, Lee et al [8] showed that patient with baseline eGFR < 60 ml/min/1.73 m^2^ had 43% higher chance of developing stroke events than those with baseline eGFR > 90 ml/min/1.73 m^2^; however, they did not find significant increase risk of stroke among patients with eGFR of 60-90 ml/min/1.73 m^2^. They also demonstrated that the effect of reduced eGFR was more profound on risk of fatal stroke than all other types of stroke, which is in line with the fact that kidney disease even with mild severity is independently associated with poor prognosis in patients with stroke disease resulting in higher risk of death [8]. It has been also measured that those with low GFR had smaller brain volume, smaller deep white matter volume and more white matter layer and these associations were independent of cardiovascular risk factors [21].

Hypertension is known to be the single most important risk factor for all kinds of stroke. In an overview of reviews, Lawes et al documented a continuous steep and log linear association between blood pressure and stroke [22]; they also highlighted a 10 mmHg lower SBP or 5 mmHg lower DBP to be associated with 30-40% lower risk of stroke. In the current study, among modifiable and non-modifiable risk factors, hypertension showed the greatest PAF for total as well as ischemic stroke events.

Consistent with previous studies, in our population, diabetes independently increased the risk of stroke event. The PAF of 24% of diabetes for ischemic stroke in the current study was in the range of 5–27% reported in Goldstein et al study [6]. Despite the significant risk of diabetes for stroke events, no strong benefit in stroke reduction with tight glycemic control has been shown [23]. However, control of blood pressure in patients with type 2 diabetes as part of a complete cardiovascular risk-reduction program and treatment of adults with diabetes with a statin, especially those with additional risk factors, is recommended to lower risk of a first stroke [24].

In the current study, smoking showed 73% risk for incident ischemic stroke, which was marginally significant and leading to PAF of 14% for incident stroke. Similarly, Goldstein et al recently reported that among risk factors for incident stroke, smoking showed a PAF of 12 to 14% [6]. A meta-analysis of 32 studies reported the relative risk for ischemic stroke to be 1.9 (95% CI, 1.7 to 2.2) for smokers compared with nonsmokers [25].

In this study, we failed to detect any relationship between high TG, low HDL and hypercholesterolemia with stroke events. There are still ongoing debates about the role of lipid components in the risk of stroke event and epidemiologic findings are not consistent [26]. Recently, a meta-analysis of 61 prospective studies, showed a weak association between lipid profiles and stroke events [27]. They found that the association between serum cholesterol and stroke mortality was modified by age and blood pressure levels, whereas no association was observed between serum cholesterol and stroke, except in individuals aged 40-59y and in participants with normal or high normal blood pressure. Also, most but not all the epidemiologic studies have found a reverse association between hemorrhagic stroke and level of blood cholesterol [28].

An important strength of our study was that, we used a population based cohort study to determine the risk factors of stroke and reached a model with good fitness in our analysis, indicating that we had considered the main risk factors in our models with acceptable low residuals; some of our limitations however, merit mentioning; first due to the limited number of our stroke events, we were unable to separately analyze and assess the risk factors for hemorrhagic stroke. Wu et al [29] had concluded that the risk factors of stroke subtypes differ as they had have different etiopathologies. Second, in the current study, also as reported from other studies in Iran [3,9], we might fail to detect all cerebrovascular events in district 13 of Tehran because there are different types of public and private healthcare systems in Iran, and the public hospitals are not completely free of charge; hence some of people who do not have any type of healthcare insurance might being managed in outpatient clinics and not be admitted to hospital. Furthermore, despite a referral healthcare structure in Iran, there may be situations where people bypass a primary care contact and self-refer to private specialists [3,9]. Third, we analyzed the associations between risk factors and stroke events from a single measurement at baseline, which may have misclassified the risk factor profiles of some individuals; contributing to the attenuation between the risk factors and incident stroke.

## Conclusions

In a population based study in Iran, we have demonstrated that among modifiable risk factors, CKD as well as hypertension, diabetes and smoking are the strongest independent predictors of ischemic stroke. Targeted interventions that prevent hypertension, CKD, and diabetes and promote smoking cessation in current smokers could largely lessen the burden of stroke events in Iran.

## Competing interests

The authors declare that they have no competing interests.

## Authors’ contributions

NF participated in the conception and design of the study, statistical analysis, interpretation of data and drafting the manuscript. DK participated in statistical analysis, drafting the manuscript and interpretation of data. RM participated in statistical analysis, drafting the manuscript and interpretation of data. FA and FH participated in its design and coordination and revised the manuscript for important intellectual content. All authors read and approved the final manuscript.

## Pre-publication history

The pre-publication history for this paper can be accessed here:

http://www.biomedcentral.com/1471-2377/12/117/prepub
